# MiRNA-loaded MSC exosomes restore autophagy flux for acute pancreatitis therapy

**DOI:** 10.3389/fimmu.2025.1613716

**Published:** 2025-08-06

**Authors:** Haojie Zeng, Tonghua Wu, Si Luo, Anxiang Zeng

**Affiliations:** ^1^ The First School of Clinical Medicine, Guangdong Medical University, Zhanjiang, Guangdong, China; ^2^ Meizhou Clinical Medical College, Guangdong Medical University, Meizhou, Guangdong, China; ^3^ Meizhou People’s Hospital, Meizhou, Guangdong, China

**Keywords:** acute pancreatitis, autophagy, miRNA, exosomes, signaling pathway

## Abstract

Acute pancreatitis (AP) is an unpredictable and potentially fatal disease. Currently, it is believed that the pathological mechanism of AP is closely related to autophagy imbalance, abnormal activation of inflammatory signals, and impairments in cell damage repair. Autophagy exhibits a double-edged sword effect of “activation accompanied by flux impairment” in AP. In this article, a systematic review is conducted on how mesenchymal stem cells (MSCs) and their secreted exosomes deliver functional miRNAs, targeting and regulating pathways such as PI3K/AKT/mTOR to achieve multiple effects including anti-inflammation, regeneration promotion, and restoration of autophagy homeostasis, providing new strategies for AP treatment. Current research challenges focus on the standardization of exosome preparation, optimization of miRNA delivery efficiency, and long-term safety evaluation. Further elucidation of the “cell-vesicle-miRNA-target pathway” cascade network, combined with multi-omics technology to develop precise intervention programs, is needed to advance AP treatment from mechanistic exploration to clinical translation.

## 1 Introduction

Acute pancreatitis is an inflammatory disease of the pancreatic exocrine glands associated with tissue damage and necrosis (1). The core pathophysiology of this disease involves abnormal trypsin activation, leading to autodigestion of acinar cells, which subsequently triggers amplification of a cascade of localized inflammatory responses. In severe cases, it can progress to systemic inflammatory response syndrome (SIRS) and multi-organ failure (2). Common clinical factors contributing to the disease include biliary system disorders (such as cholelithiasis), alcoholism, traumatic injury, drug toxicity, and autoimmune disorders (**Table 1**).

**Table 1:** Causes of AP.

Etiology	Pathogenesis
Gallstone	(1) Due to choledocholithiasis incarcerated in the nipple (the confluence of pancreatic duct and bile duct), which subsequently triggers pancreatic juice outflow obstacle, leading to the activation of pancreatic enzymes within the pancreatic duct; (2) Inflammation associated with cholangitis directly spreads to the pancreas (3).
Hypertriglyceridemia	(1) Unsaturated fatty acids cause acinar cell necrosis by releasing intracellular calcium, promoting inflammatory responses; (2) Hyperlipidemia induces pancreatic microcirculation disturbances; (3) Calcium ion overload and endoplasmic reticulum stress; (4) Oxidative stress; (5) Genetic polymorphism (4).
Alcohol abuse	The non-oxidative metabolism of ethanol produces fatty acid ethyl esters (FAEEs), which accumulate in mitochondria and undergo hydrolysis, disrupting intracellular calcium ion homeostasis. Extracellular calcium ion overload inhibits mitochondrial function by opening the mitochondrial permeability transition pore (MPTP), and ATP production is affected, thereby activating the necrotic cell death pathway (5).
Endoscopic Retrograde Cholangiopancreatography (ERCP)	ERCP involves a combination of chemical, thermal, mechanical, hydrostatic, enzymatic, allergic, and microbial injuries resulting from instrumentation of the papilla and/or hydrostatic injury due to overfilling of the pancreatic duct with contrast media. The influence of these factors triggers a sequence of events leading to premature activation of pancreatic proteolytic enzymes, autodigestion, and the release of inflammatory cytokines that cause both local and systemic effects (6).
Drug toxicity	Most of the proposed mechanisms of drug-induced injury remain unconfirmed, but the underlying mechanism may be attributed to a specific reaction (such as hypersensitivity or the accumulation of toxic metabolites) rather than intrinsic toxicity (7).
Autoimmune Pancreatitis (AIP)	(1) Type 1 AIP is an IgG4-related disease dominated by a Th2-type immune response, potentially associated with gene polymorphisms such as HLA-DRB1*0405 and FCGR2B; (2) Type 2 AIP is idiopathic duct-centric pancreatitis dominated by a Th1-type immune response, with fewer IgG4-positive cell infiltrations, and may be related to neutrophil extracellular traps (NETs) and cytokine storms (8).

Acute pancreatitis, one of the gastrointestinal diseases with the highest hospitalization rates globally, is characterized by sudden severe abdominal pain accompanied by multi-organ dysfunction. It carries the potential risk of progressing to pancreatic necrosis and persistent organ failure, exhibiting an unpredictable and potentially fatal course (9). Based on the Revised Atlanta Classification (RAC), the severity of AP can be categorized into three levels: mild acute pancreatitis, moderately severe acute pancreatitis, and severe (SAP). This classification is determined by factors such as the duration of organ failure (transient/persistent) and the presence of local or systemic complications (10). From a cellular mechanism perspective, the functional homeostasis of pancreatic acinar cells (PAC) relies on the precise coordination of organelles like the endoplasmic reticulum, mitochondria, and lysosomes. Dysfunction in these organelles can disrupt the metabolic balance within PACs, triggering the pathological process of pancreatitis and leading to cell death. Although approximately 80% of acute pancreatitis (AP) cases exhibit a self-limiting course, 20% of patients still progress to moderately severe acute pancreatitis or severe acute pancreatitis (SAP), complicated by systemic inflammatory response syndrome (SIRS) and multi-organ failure, with a mortality rate as high as 39% (11). Survivors frequently suffer from severe short-term complications and long-term organ damage. Currently, there are no internationally approved specific drugs for acute pancreatitis and its complications. Existing treatments primarily involve intravenous fluid resuscitation, analgesia, enteral nutrition, protease inhibitors, and other combination therapies. Intensive care, organ support, parenteral nutrition, antibiotics, and pancreatic exocrine and endocrine replacement therapies may be necessary in severe cases (12). Unfortunately, these treatments, due to their lack of specificity, often have limited efficacy and may be associated with adverse reactions, resulting in poor patient outcomes (13). Hence, the development of novel diagnostic and therapeutic approaches has become an urgent need in current research.

Recent research has focused on the mechanism of autophagy regulation in AP. As a core catabolic pathway for maintaining cellular homeostasis, autophagy exerts a protective effect on pancreatic cells by eliminating damaged organelles (14). Genetic studies have confirmed that defects in genes encoding autophagy-related proteins (such as ATG5 and ATG7) or lysosome function-related proteins (such as LAMP2) can disrupt the autophagic flux in acinar cells, thereby inducing pancreatitis (15). It is noteworthy that in the mammalian target of rapamycin (mTOR) signaling pathway, the PI3K/AKT/mTOR axis plays a pivotal role, particularly mTOR complex 1 (mTORC1), which regulates the initiation of autophagy in acute pancreatitis (AP) through ULK1 phosphorylation (16). Dysregulation of this signaling axis promotes trypsinogen activation, thereby triggering an inflammatory cascade (17). The dynamic regulatory mechanism of autophagy activity exerts crucial protective effects on the pancreas, providing novel therapeutic targets for AP.

**Figure 1:**
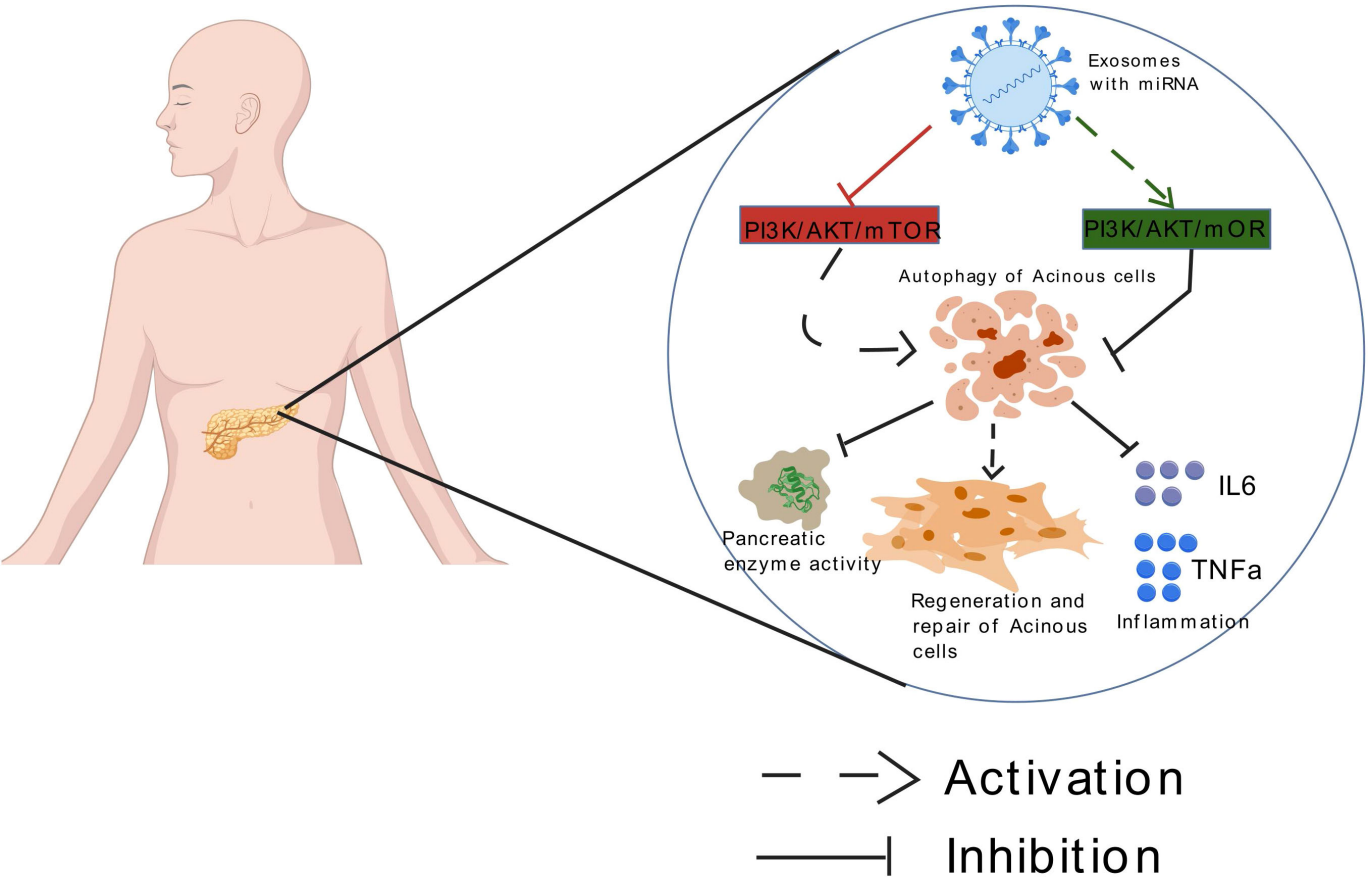
Exosomes loaded with miRNA can mediate autophagy of acinar cells by targeting the mTOR signaling pathway, thereby improving acute pancreatitis.

In the autophagy regulatory network, microRNAs (miRNAs) represent a class of small, evolutionarily conserved non-coding RNA molecules that mediate post-transcriptional gene silencing by binding to the 3’ untranslated region (UTR) of target mRNAs. They are widely involved in cellular processes such as development, differentiation, and apoptosis (18). Research indicates that miRNAs participate in almost every stage of autophagy, including critical steps like phagophore formation, autophagosome maturation, and lysosomal degradation (19). Meanwhile, MSCs therapy exhibits unique advantages in the treatment of AP due to its multipotent differentiation capacity and paracrine characteristics (20). MSCs not only directly repair and replace damaged tissues (21) but also secrete anti-inflammatory factors to inhibit apoptosis and fibrosis (22). The exosomes secreted by MSCs, with their low immunogenicity, long-lasting circulation, and ability to penetrate the blood-brain barrier, serve as ideal drug delivery vehicles (23). These nanovesicles efficiently carry miRNAs, proteins, and immunomodulatory factors, providing a technical platform for targeted modulation of the PI3K/AKT/mTOR pathway. Based on these breakthroughs, this article systematically elucidates the molecular mechanism of exosome-delivered miRNA in regulating the PI3K/AKT/mTOR pathway in AP, aiming to provide an innovative theoretical framework and transformation strategy for precise disease diagnosis and treatment **Figure 1**.

## 2 Pathophysiology of acute pancreatitis

Acute pancreatitis is a disease characterized by acute inflammation of the pancreatic parenchyma, which in severe cases can lead to local tissue destruction and multiple organ dysfunction (24). Its pathophysiology exhibits a multidimensional interaction, involving crucial pathological processes such as abnormal activation of trypsinogen, inflammatory cascade reactions, metabolic imbalances, and cell death. These factors collectively contribute to pancreatic tissue damage and dysfunction.

### 2.1 Abnormal activation of trypsinogen: the central trigger of pathological cascade

The premature activation of trypsinogen serves as a key initiating event in the onset of AP. Under physiological conditions, trypsinogen is stored in an inactive form within pancreatic acinar cells and becomes proteolytically active upon activation by enterokinase (25). However, when pancreatic duct obstruction or direct damage to pancreatic acinar cells occurs due to factors such as gallstones, alcohol, drugs, or metabolic disturbances, lysosomal hydrolase cathepsin B (CTSB) abnormally fuses with zymogen granules. This fusion catalyzes the conversion of trypsinogen into active trypsin, initiating pancreatic autodigestion and inflammatory responses (26). This process constitutes the central triggering mechanism in the pathological development of AP.

### 2.2 Cascade amplification of immune and inflammatory responses

Abnormal trypsin activation triggers a dual inflammatory response: (1) Local damage promotes neutrophil infiltration and the release of neutrophil extracellular traps, which further activate trypsinogen through mechanisms such as STAT-3 and MMP-9, forming a vicious positive feedback loop (27). (2) Macrophages polarize towards a proinflammatory M1 phenotype under the stimulation of damage-associated molecular patterns (DAMPs), releasing inflammatory mediators such as TNF-α and IL-1β through the NF-κB pathway, which intensifies local inflammation and spreads systemically, inducing systemic inflammatory response syndrome (SIRS) (28). Notably, although M2 macrophages exhibit anti-inflammatory and tissue healing properties, their polarization is significantly reduced or impaired during the acute phase of AP (29, 30). This results in an imbalance between pro-inflammatory (M1) and anti-inflammatory/repair (M2) responses, further aggravating inflammation and tissue injury.

### 2.3 Pathological calcium signaling

Intracellular calcium ions (Ca^2+^) serve as key signaling molecules that are widely involved in the regulation of cellular functions (31). Under physiological conditions, the calcium pump system maintains intracellular Ca^2+^ homeostasis through dynamic regulation (32). In acute pancreatitis, pathogenic factors such as bile acids and ethanol metabolites disrupt Ca^2+^ homeostasis through a dual mechanism: (1) activating endoplasmic reticulum calcium channels to trigger abnormal Ca^2+^ release; (2) continuously activating store-operated calcium channels, leading to persistent extracellular Ca^2+^ influx (33, 34). Calcium Overload Exacerbates Injury Through the Following Mechanisms: (1) Inhibition of mitochondrial complex V function, leading to impaired ATP synthesis (35). (2) Impairment of sarcoplasmic/endoplasmic reticulum calcium ATPase (SERCA)-mediated cytosolic Ca^2+^ clearance: On one hand, elevated Ca^2+^ concentrations inhibit SERCA activity via oxidative stress. On the other hand, ATP synthesis dysfunction results in an insufficient energy supply for SERCA function, further compromising its Ca^2+^ clearance capacity. This establishes a vicious cycle of “calcium overload–energy depletion” (36, 37). (3) Direct activation of the necroptosis pathway: Calcium overload binds and activates calmodulin (CaM), subsequently activating Ca^2+^/calmodulin-dependent protein kinase II (CaMKII). Activated CaMKII phosphorylates receptor-interacting protein kinase 3 (RIPK3), promoting the formation of the necrosome. This ultimately triggers phosphorylation, oligomerization, and pore formation by mixed-lineage kinase domain-like protein (MLKL), leading to plasma membrane rupture and necrotic cell death (38).

### 2.4 Mitochondrial dysfunction and energy metabolism crisis

Mitochondria exhibit characteristic pathological changes in AP. These include: (1) sustained opening of the mitochondrial permeability transition pore (MPTP) leading to the collapse of membrane potential; (2) an outbreak of reactive oxygen species (ROS) triggering lipid peroxidation and protein damage; (3) impediment of the tricarboxylic acid cycle, exacerbating ATP synthesis deficiency (39, 40). This energy crisis not only impairs cellular repair capabilities but also accelerates programmed cell death by releasing apoptotic factors such as cytochrome C.

### 2.5 Dysfunction of the autophagy-lysosome system

Physiological autophagy plays a protective role in the early stages of AP, maintaining homeostasis by eliminating damaged organelles and misfolded proteins (41, 42). However, in cases of severe pancreatic injury, lysosomal dysfunction and the resulting blockade of autophagic flux play pivotal roles in the pathological progression of pancreatitis. Impaired lysosomal function manifests as: (1) loss of membrane stability (e.g., disruption of membrane integrity by alcohol metabolites or bile acids), (2) failure to maintain acidity (elevated pH due to V-ATPase dysfunction), and (3) reduced hydrolytic enzyme activity. These impairments lead to abnormal leakage of cathepsins into the cytoplasm, subsequently activating trypsinogen and triggering pancreatic acinar cell autodigestion (43, 44). Concurrently, this process induces autophagic flux blockade: although autophagosomes can form (evidenced by LC3-II accumulation), impaired lysosomal fusion or degradation capacity results in accumulation of autophagic substrates such as p62, preventing clearance of damaged organelles and misfolded proteins (45). This blockade further amplifies oxidative stress and endoplasmic reticulum stress, promoting the release of inflammatory cytokines including IL-1β and TNF-α (46). Clinical studies have confirmed that downregulation of lysosomal membrane protein (LAMP-2) expression in pancreatic tissues of severe acute pancreatitis patients is closely associated with abnormal elevation of the autophagy marker p62, highlighting the core pathological axis of “lysosomal stability disruption–autophagic degradation failure–inflammatory cascade” (47).

### 2.6 Systemic responses and complications

Approximately 20% of patients with AP progress to a severe form, essentially characterized by the uncontrolled expansion of local inflammation to a systemic level. This involves: (1) an inflammatory mediator storm triggering capillary leak syndrome; (2) pancreatic necrotic products entering the liver via the portal vein, exacerbating oxidative stress; (3) myocardial depressant factor leading to circulatory failure. These pathological processes can ultimately result in multiple organ dysfunction syndrome, constituting the primary cause of death (48).

## 3 The role of autophagy in acute pancreatitis

### 3.1 Overview

Autophagy is an evolutionarily conserved cellular degradation and recycling process that plays a critical role in maintaining cell survival and function by eliminating and recycling damaged or dysfunctional organelles, abnormal protein aggregates, and regulating endoplasmic reticulum homeostasis (41). Based on mechanistic differences, autophagy can be classified into three types: microautophagy, macroautophagy, and chaperone-mediated autophagy (CMA). Macroautophagy involves the formation of double-membrane autophagosomes that engulf large cytoplasmic components (e.g., damaged organelles or protein aggregates) and deliver them to lysosomes for degradation. Microautophagy entails the direct engulfment of cytoplasmic components through invagination of the lysosomal membrane itself. Chaperone-mediated autophagy involves the selective translocation of specific cytosolic proteins into lysosomes via recognition by lysosomal membrane receptors, without the formation or significant remodeling of membrane structures (49). Among them, macroautophagy is the most extensively studied type of autophagy. Its core process involves the formation of a double-membrane structure called the autophagosome in the cytoplasm, which then fuses with lysosomes to form autolysosomes, where the degradation and recycling of contents are completed by lysosomal hydrolases (49). In summary, the process of autophagy (specifically macroautophagy) can be outlined in four main steps: (1) Initiation: Under conditions of nutrient deprivation or cellular stress, the energy/nutrient-sensing kinase AMPK is activated while mTORC1 is inhibited. These two kinases antagonistically regulate the activation of the unc-51-like autophagy activating kinase 1 (ULK1) complex, thereby triggering the formation of autophagosome precursors; (2) Elongation: Autophagy-related gene (ATG) proteins mediate the elongation of the autophagosome membrane. Microtubule-associated protein 1 light chain 3 (LC3) undergoes lipidation (conversion from LC3-I to LC3-II) and anchors to the membrane surface, driving autophagosome maturation; (3) Fusion: The mature autophagosome fuses with the lysosome through lysosome-associated membrane proteins (LAMPs) to form the autolysosome; (4) Degradation: Acidic hydrolases within the lysosome (such as CTSB and CTSL) break down the contents, releasing small molecules for cellular reuse (50). Autophagy flux is a key indicator for measuring cellular autophagy function, which reflects the rate at which autophagy substrates within cells are degraded and recycled over a period of time, covering the dynamic processes of autophagosome formation, fusion with lysosomes, and substrate degradation. Cells can precisely regulate autophagy flux by modulating the mTOR and AMPK signaling pathways, TFEB and FOXO transcription factors, thereby ensuring cellular homeostasis and metabolic balance (51). It’s worth noting that the completion of autophagic flux highly depends on lysosome function, including the efficiency of autophagosome-lysosome fusion and lysosome enzyme activity. Therefore, physiologically self-activated autophagy needs to maintain a dynamic balance between autophagosome generation and lysosome degradation capacity (52).

### 3.2 Pathological role of autophagy dysfunction in AP

Autophagy plays a dual role in the occurrence and development of AP, exhibiting both protective and damaging effects. On one hand, during the pathological process of pancreatitis, cells are subjected to various stress factors. Autophagy maintains cell survival and function by eliminating damaged mitochondria and misfolded proteins, reducing endoplasmic reticulum stress, and inhibiting inflammatory cascade reactions (53). However, the regulation of autophagy is a complex process, and excessive activation of autophagy can lead to autophagy-dependent cell death and the release of proinflammatory mediators, exacerbating tissue damage (54). Research indicates that AP does not block autophagosome formation but disrupts autophagy flux through the following mechanisms: (1) In the early stages of autophagy, due to certain reasons, excessive autophagosome generation leads to the accumulation of a large number of undegraded autophagosomes within cells, triggering cell death and tissue damage (55). (2) In later stages, lysosomal dysfunction (such as imbalances in cathepsin activity and reductions in LAMP proteins) prevents the fusion of autophagosomes and lysosomes, resulting in the inadequate clearance of autophagosomes. This further intensifies autophagosome retention, mediating two key pathological responses: vacuolization of acinar cells and the accumulation of intra-acinar trypsin (56). In summary, pancreatitis has two primary effects on autophagy: autophagy is activated, but its flux is impaired/delayed.

The typical pathological feature of AP is the abnormal accumulation of large vacuoles within acinar cells. Immunohistochemical analysis reveals that these vacuoles exhibit co-positive expression of the autophagosome marker LC3 and the lysosomal marker protein LAMP, suggesting that their formation mechanism may involve increased autophagosome formation, decreased lysosomal degradation, or a combination of both (57). As a core regulatory factor for autophagy initiation, Beclin-1 triggers the formation of autophagosome precursors by mediating the assembly of class III phosphatidylinositol 3-kinase (PI3K) complexes (58). LC3 serves as a central biomarker for autophagy flux, and an elevated LC3-II/I ratio significantly correlates with an increased number of autophagic vacuoles, reflecting an imbalance between autophagosome generation and degradation (59). The level of P62 protein (SQSTM1), an autophagy substrate adaptor protein, increases in response to impaired autophagic degradation (60). Mechanistic studies indicate that downregulation of AMPK/SIRT1 signaling axis activity during AP leads to abnormal accumulation of Beclin-1, impeded P62 degradation, and an elevated LC3II/I ratio, collectively exacerbating autophagy flux blockage and inflammatory cascade reactions (61). Notably, activating AMPK can restore autophagy homeostasis by upregulating SIRT1, presenting a potential therapeutic target. LAMPs play a critical role in autophagy flux completion by maintaining lysosomal membrane integrity, regulating autophagosome-lysosome fusion, and mediating acidic hydrolase activity (62). Lysosomal dysfunction, manifested by LAMP degradation, is a common occurrence in various experimental models and human pancreatitis. Among them, LAMP-2 is crucial for acinar cell function, and its deficiency directly disrupts autophagosome-lysosome fusion, representing a key mechanism underlying autophagy flux impairment in AP (15).

Cathepsins are a class of lysosomal proteases that exhibit diverse functions in different parts of the cell, playing crucial roles in intracellular protein degradation, energy metabolism, and extracellular matrix degradation (63). Cathepsin L (CTSL) is capable of degrading both trypsin and trypsinogen, while cathepsin B (CTSB) effectively converts trypsinogen into active trypsin (64). Research indicates that during pancreatitis, an increase in CTSB activity promotes abnormal activation of trypsinogen, whereas impaired CTSL activity leads to inadequate degradation of pancreatic enzymes. The imbalance between these two activities exacerbates the accumulation of pancreatic enzymes within the acinar cells, with the effects of CTSL impairment being particularly significant (65).

The impairment of autophagy primarily manifests as a disruption in the integrity of the dynamic autophagic flux process, which can occur at any step of autophagy, including decreased or defective autophagosome formation, impeded fusion with lysosomes, or reduced lysosomal proteolytic enzyme activity (i.e., lysosomal dysfunction) (66). In AP, the central feature of autophagy dysregulation is “autophagy activation with impaired flux,” specifically characterized by increased LC3-II/I, decreased LAMP, and the accumulation of P62. This process contributes to the pathological progression of pancreatitis through mechanisms such as lysosomal dysfunction, imbalance in pancreatic enzyme metabolism, and oxidative stress. Studies have shown that mice with pancreas-specific knockout of autophagy-related proteins Atg5 or Atg7 exhibit p62 accumulation, mitochondrial dysfunction, and exacerbated oxidative stress, ultimately leading to increased cellular stress, necrosis, inflammation, and fibrosis (67, 68).

## 4 Major pathways regulating autophagy in acinar cells

Abnormal autophagy in acinar cells during AP is closely associated with various signaling pathways (**Table 2**), such as the AMPK/SIRT1 signaling pathway (61, 69), the PI3K/AKT/mTOR signaling pathway (17), the AKT/AMPK/mTOR signaling pathway (70), the Beclin-1 signaling pathway (71), the JAK/STAT3 signaling pathway (72), and the NF-kB/TNFα/SIRT1 signaling pathway (73). Protein kinase B (AKT) is downstream of phosphatidylinositol 3-kinase (PI3K) and plays an antagonistic role in autophagy, involving the activation of the mTORC1 complex and the inhibition of ULK1 and ATG13 to block the initiation of autophagy (74). Mammalian target of rapamycin (mTOR), as a “metabolic sensor,” blocks autophagy by inhibiting the ULK1 complex when cellular energy is sufficient, serving as a key negative regulator of autophagy (75). Among these, the regulatory roles of PI3K/AKT and mTOR have been widely recognized and are considered key molecules in autophagy (76). Therefore, we will focus on the effects of PI3K/AKT/mTOR on AP in the following discussion.

**Table 2:** Effects of different signaling pathways on pancreatic autophagy and AP.

No.	Correlation pathway	Influence on the course of the disease	References
1.	AMPK/SIRT1	During AP, there is a decrease in AMPK and SIRT1, while p62, Beclin-1, and LC3 II/I increase, indicating impaired autophagy.	(61, 69)
2.	PI3K/AKT/mTOR	Xanthohumol may inhibit the AKT/mTOR pathway by suppressing IL-17, thereby improving autophagy (increased autophagic flux), reducing oxidative stress, and treating SAP.	(17)
3.	AKT/AMPK/mTOR	H_2_S excessively activates autophagy through the AKT/AMPK/mTOR pathway, exacerbating the pathological process of AP.	(70)
4.	lncRNA-PVT1/miR-30a-5p/Beclin-1	Inhibition of lncRNA-PVT1 expression suppresses autophagy by targeting the miR-30a-5p/Beclin-1 signaling axis, protecting pancreatic acinar cells from damage during SAP.	(71)
5.	JAK/STAT3	Astaxanthin inhibits pancreatic damage in AP by targeting the IL-6/JAKs/STAT3 signaling axis-mediated apoptosis and autophagy.	(72)
6.	NF-kB/TNFα/SIRT1	Treatment with Picroside II downregulates TNF-α and SIRT1 by inhibiting NF-κB expression, reducing autophagic activity in SAP, and thereby improving the condition.	(73)

The PI3K/AKT/mTOR signaling pathway plays a pivotal role in regulating inflammatory responses and autophagy. Studies have indicated that both inhibition and activation of this pathway can improve the pathological process of AP, but the therapeutic efficacy depends on the spatiotemporal specificity of the target and regulatory mechanism. Multiple studies have demonstrated that inhibiting the PI3K/AKT/mTOR signaling pathway can activate autophagy, suppress trypsinogen activation, reduce tissue damage, and hinder inflammatory progression, thereby improving AP. For instance, research has found that phillygenin (PHI) and xanthohumol can restore impaired autophagic flux by inhibiting the PI3K/AKT/mTOR signaling pathway, reducing p62 levels, and increasing LAMP-2 levels, ultimately improving AP and preventing the progression and deterioration of SAP (17, 47). Yang et al. discovered that Chaiqin Chengqi Decoction (CQCQD) attenuates the severity of alcohol-induced AP by activating the antioxidant protein response and downregulating the PI3K/Akt signaling pathway in the pancreas and visceral adipose tissue (77). Wortmannin significantly reduces the redistribution of CTSB by inhibiting PI3K, effectively preventing intrapancreatic activation of trypsinogen *in vivo* (78). PI3K/Akt inhibitors also lower the expression of inflammatory cytokines in SAP rats, exerting potent anti-inflammatory and antioxidant effects by inhibiting NF-kB nuclear translocation and downregulating the transcription of NF-kB-dependent pro-inflammatory genes, including TNF-α, IL-1β, and IL-6 (77, 79–82).

However, another set of studies indicates that activating the PI3K/AKT/mTOR signaling pathway under specific conditions to inhibit autophagy can also play a role in improving AP. Macrophages are the most abundant immune cells in the regenerating pancreas (83). The regeneration process of exocrine acinar cells involves a transient phase of inflammation, acinar-to-ductal metaplasia (ADM), and acinar redifferentiation (84). Activation of PI3K-AKT in macrophages promotes inflammation resolution during the ADM phase, improving pancreatic regeneration and organ function recovery (85, 86). Studies have found that IGF-1 (insulin-like growth factor, a PI3K agonist), MZB1 (marginal zone B and B-1 cell-specific protein 1), and rhubarb inhibit autophagy by activating the PI3K/AKT/mTOR signaling pathway, stimulate pancreatic cell proliferation, induce acinar ADM and redifferentiation, and accelerate pancreatic repair (87–89). It’s worth noting that IGF-1 can also significantly reduce the expression of proinflammatory cytokines such as IL-6 in a dose-dependent manner, exhibiting the same anti-inflammatory effect as the PI3K inhibitor wortmannin. Li et al. (90) found that ulinastatin has anti-inflammatory and antioxidant functions in LPS (lipopolysaccharide)-treated RAW264.7 cells, and its protective effect can be attributed to the activation of the PI3K/Akt-Nrf2 axis and the inhibition of the Thr183p/JNK/NF-κB axis. Wang et al. (91) found that LXA4 (lipoxin A4) activates the PI3K/Akt and PKC pathways, induces Nrf2 phosphorylation, leads to the upregulation of HO-1, reduces cell adhesion, and protects mitochondrial function, exerting a cytoprotective effect in lung injury caused by SAP. Research shows that spautin-A41, as a novel and effective autophagy inhibitor, appears to have a therapeutic effect on AP, and its mechanism may be to reduce the expression level of the PI3K complex, thereby inhibiting acinar trypsin activation and inflammatory response (92).

Indeed, numerous studies have established a close relationship between AKT-regulated autophagy and inflammatory responses. Autophagy can negatively regulate inflammatory reactions by eliminating damaged mitochondria and aggregated inflammatory signaling proteins, thereby reducing the production of reactive oxygen species (ROS) (93). In fact, inflammation and autophagy are mutually causal, and a chronic inflammatory environment can also lead to impaired autophagy function (94). Various studies have demonstrated that inflammatory factors can inhibit ATG gene expression and disrupt autophagosome-lysosome fusion through the activation of the NF-κB pathway, ultimately suppressing autophagy (95). Therefore, during the acute phase of AP, the PI3K/AKT/mTOR signaling pathway can be inhibited to activate autophagy, clear damaged organelles, and suppress trypsin activation. Conversely, during the recovery phase, activating this pathway promotes acinar regeneration and inflammation resolution.

## 5 The regulatory role of miRNAs in AP

### 5.1 Biological functions and therapeutic potential of miRNAs

**Figure 2:**
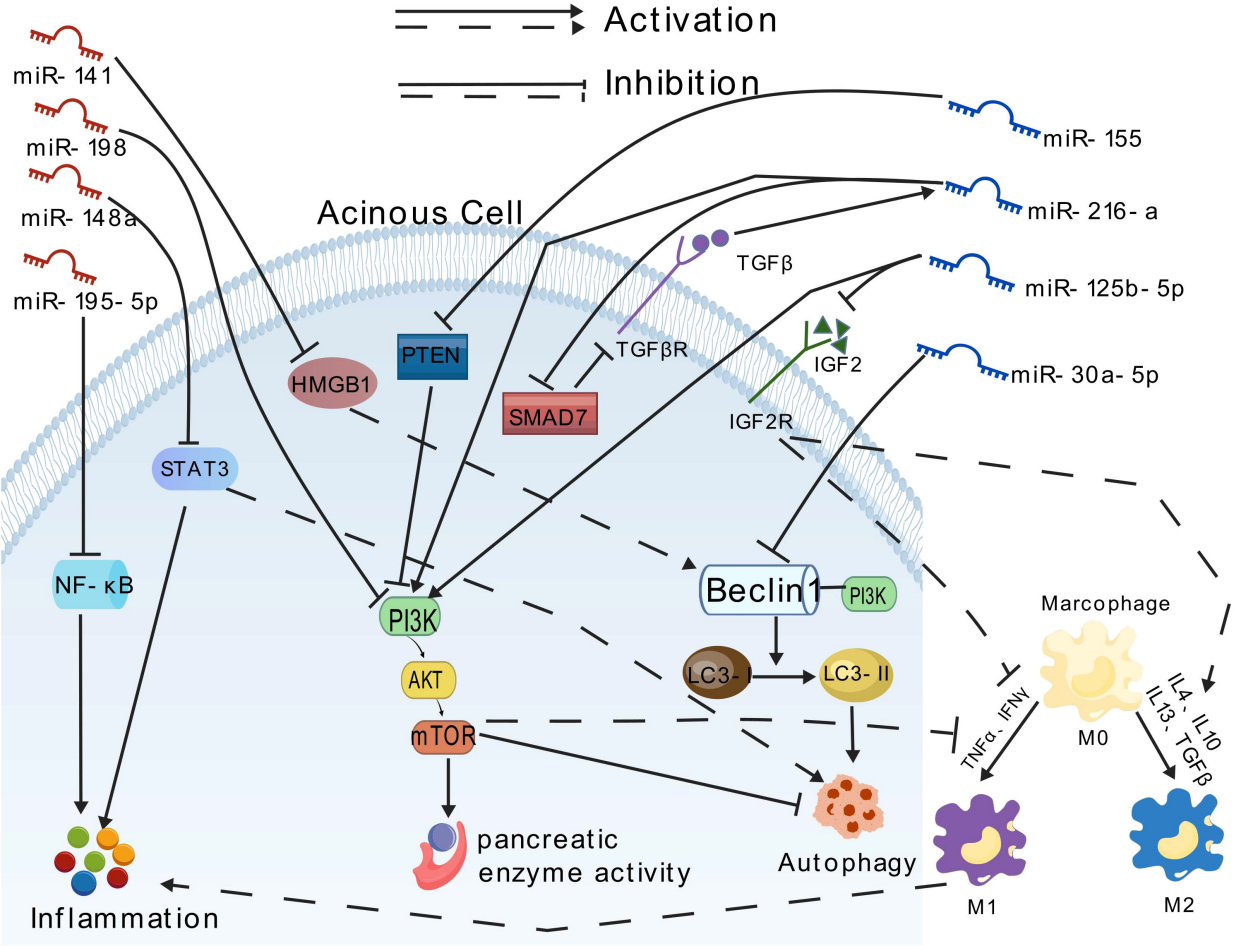
Effects of different miRNAs on pancreatic blast cells.

The miRNAs play a pivotal role in the regulation of gene expression. Therefore, modulating miRNA function *in vitro* and *in vivo* represents a potential therapeutic approach to regulate disease pathophysiology at the genetic level. Recently, increasing evidence has demonstrated the diverse functional roles of miRNAs in acinar cell injury, inflammation, and distant organ dysfunction (**Table 3**; **Figure 2**).

**Table 3:** Effects of different miRNAs on autophagy and AP in pancreas.

MiRNA	Correlation pathway	MiRNA′s effect on pathways	Influence on the course of the disease	Reference
miR-30a-5p	miR-30a-5p/Beclin-1/PI3K	Activation	Inhibiting autophagy to exacerbate pancreatitis	(71)
miR-195-5p	NF-κB/TNF-α	Activation	Eliminating inflammatory damage in the pancreas	(103)
miR125b-5p	PI3K/AKT/IGF2	Activation	Promoting M1 macrophage polarization and the release of inflammatory cytokines, exacerbating AP (acute pancreatitis)	(100)
miR-216a	PI3K/Akt, TGF-β-PTEN-Smad7	Activation	Aggravating AP	(102)
miR-148a	IL-6/STAT3	Activation	Reducing IL-6 mRNA and protein levels, decreasing autophagosomes and autolysosomes, improving AP	(104, 105)
miR-194	MALAT1-MicroRNA-194-YAP1 Pathway	Activation	Lowering the secretion of IL-6 and TNF-α, ameliorating AP	(106)
miR-141	HMGB1/beclin-1	Activation	Suppressing autophagy to ameliorate AP	(107)
miR-155	PI3K/AKT/mTOR	Activation	Targeting the PI3K/AKT/mTOR signaling pathway, promoting the formation of autophagosomes, impairing autophagy flux, and facilitating inflammation, thus aggravating AP	(96–99)
miR-198	miR-198/PI3K/Akt	Activation	Inhibiting apoptosis of pancreatic cells in rat models, providing conditions for pancreatitis recovery	(109)
miR-20b-5p	miR-20b-5p/AKT3	Activation	Enhancing autophagy, suppressing inflammation and apoptosis in pancreatic acinar cells, and promoting angiogenesis to prevent damage to pancreatic tissue in SAP (severe acute pancreatitis)	(108)

### 5.2 Pathological mechanisms of pro-inflammatory miRNAs

Some miRNAs exhibit pro-inflammatory effects. For instance, Hu et al. (71) demonstrated that miR-30a-5p exacerbates pancreatic acinar cell injury during SAP by inhibiting autophagy through targeting the Beclin-1 gene. Beclin-1, a core regulatory factor for autophagy initiation, recruits LC3 by interacting with the PI3K complex. miR-155, recognized as the first miRNA associated with immune regulatory functions, has since been increasingly studied for its regulatory roles related to inflammatory responses. Reports indicate that miR-155 may target the PI3K/AKT/mTOR signaling pathway, inducing excessive autophagosome formation and macrophage M1 polarization, disrupting autophagy flux homeostasis, and leading to inflammatory cascade amplification, thereby aggravating AP (96–99). Zeng et al. (100) showed that exosomes derived from acinar cell line AR42J, serving as carriers of miR-125b-5p, act on IGF2 in the PI3K/AKT signaling pathway. By inhibiting IGF2 expression, miR-125b-5p promote macrophage M1 polarization and suppress M2 polarization, resulting in the release of proinflammatory cytokines and inflammatory cascade amplification, ultimately worsening AP. PTEN (phosphatase and tensin homolog) is a novel tumor suppressor gene that directly dephosphorylates the cellular second messenger PI3K, subsequently blocking downstream signaling effectors and participating in cell apoptosis regulation (101). It has been reported that overexpression of miR-216a activates the PI3K/AKT and TGF-β pathways by targeting PTEN, activating trypsinogen, and inhibiting autophagy and apoptosis of pancreatic acinar cells, thereby accelerating the progression of AP (102).

### 5.3 Protective effects of anti-inflammatory miRNAs

Conversely, certain miRNAs exhibit anti-inflammatory properties. For instance, Chen et al. (103) reported that overexpressed miR195-5p can suppress the expression of inflammatory cytokines such as TNF-α via the NF-κB pathway, thereby halting the progression of AP. Studies have indicated that miR-148a inhibits excessive autophagy activation and inflammatory responses by modulating the IL-6/STAT3 signaling axis in both *in vitro* and *in vivo* models of cerulein-induced AP, suggesting that miR-148a is a potential candidate for AP gene therapy (104, 105). Gu et al. (106) found that overexpression of miR-194 reduces the secretion of inflammatory factors like IL-6 and TNF-α, playing a profound role in regulating AP progression. Zhu et al. (107) demonstrated that miR-141 can regulate autophagy through the HMGB1/Beclin-1 pathway, inhibiting autophagosome formation and alleviating tissue damage. Tang et al. (108) discovered that miR-20b-5p directly targets AKT3 to promote autophagy, suppress inflammation, and facilitate apoptosis and angiogenesis, emerging as a new therapeutic target for SAP. Curcumin exerts profound antioxidant, anti-inflammatory, anticancer, and antiviral effects. Studies have revealed its significant protective effects on pancreatic tissue, notably reducing edema, inflammation, hemorrhage, and necrosis in rat pancreatic tissue while inhibiting the activities of amylase and lipase. The mechanism underlying these effects may be mediated through the miR-198-PI3K/Akt signaling axis (109).

## 6 The therapeutic potential of mesenchymal stem cells in acute pancreatitis

MSCs are a type of adult stem cell characterized by their low immunogenicity and multilineage differentiation potential. These cells have demonstrated significant efficacy in the treatment of various diseases, including osteoarthritis (110), liver fibrosis (111), and myocardial infarction (112). In the context of AP, early studies have confirmed that human bone marrow-derived MSCs (BMSCs) can alleviate pancreatic damage in a rat model of AP by suppressing inflammatory responses (113). Subsequent research has further untangled the underlying mechanisms by which BMSCs improve SAP, including reducing oxidative stress (114), promoting angiogenesis (115), and inhibiting pancreatic necrosis (116). Recent studies have untangled that exosomes serve as pivotal mediators of intercellular communication and therapeutic effects. Exosomes exhibit high bioavailability, stability, and the capacity to traverse biological barriers, enabling them to deliver functional molecules (e.g., miRNAs) to distal target cells, regulate gene expression, and enhance tissue healing (117). Consequently, exosomes represent an ideal platform for miRNA delivery, effectively overcoming the challenges of low delivery efficiency and rapid degradation associated with free miRNA therapeutics. Mesenchymal stem cells (MSCs) constitute one of the primary sources of exosomes. The MSC-derived exosomes carry specific miRNAs that exert therapeutic effects by modulating key signaling pathways. For instance, Song et al. (118) demonstrated that BMSCs significantly reduce pancreatic damage in SAP by upregulating the PI3K/AKT/MTOR pathway and inhibiting the expression of autophagy key molecules Beclin-1 and LC3. Further research from the same team (119) showed that inhibiting miR-138-5p in MSCs activates the FAK/PDK1/AKT/mTOR pathway, suppressing autophagy in SAP. Additionally, knocking down miR-141-3p in MSCs promotes pancreatic cell proliferation by upregulating the expression of β-catenin, c-Myc, and cyclin D1. Song et al. (120) found that miR-29a-3p carried by MSC-derived exosomes alleviates myocardial damage in a SAP model by inhibiting the HMGB1/TLR4/AKT axis. Moreover, miR-216a-5p from MSC-derived exosomes induces the transition of macrophages from a proinflammatory M1 phenotype to an anti-inflammatory M2 phenotype by inhibiting the TLR4/NF-κB signal and activating the PI3K/AKT pathway, thereby reducing systemic inflammation (121). A recent study (122) revealed that MSC-derived exosomes maintain mitochondrial dynamics stability and enhance autophagy by delivering miR-214-3p to inhibit the PI3K/AKT/mTOR pathway, thus alleviating inflammatory responses in ulcerative colitis. This mechanism may have potential applications in the treatment of AP.

More importantly, the potential of exosomes as miRNA delivery vehicles is not limited to MSCs. A variety of cell types, including immune cells, endothelial cells, epithelial cells, and even tumor cells, can secrete exosomes carrying distinct miRNA profiles, which may similarly influence the progression of AP through analogous mechanisms (123, 124). These exosomes also exhibit favorable characteristics, such as stability, low immunogenicity, and targeted delivery potential (125), thereby expanding the therapeutic possibilities for sEV-based AP treatment strategies.

In summary, exosomes, particularly those derived from therapeutic cells such as MSCs, exert multifaceted therapeutic effects—including anti-inflammatory, anti-autophagic, and pro-regenerative activities—by delivering miRNAs to modulate key signaling pathways such as PI3K/AKT/mTOR and TLR4/NF-κB. This “cell-exosome-miRNA” cascade regulation model provides a new approach for precision treatment of AP. However, further research is needed to validate the standardized preparation of exosomes (regardless of their source), miRNA delivery efficiency, and long-term safety for clinical translation, as current related studies are limited.

## 7 Discussion

In recent years, the risk factors for acute pancreatitis have gradually increased, leading to a rise in its incidence and mortality rates. This trend is particularly concerning for severe acute pancreatitis, where conventional diagnosis and treatment often fail to prevent serious complications and recurrences. Therefore, there is an urgent need for innovative optimizations in early diagnosis, prognosis evaluation, and therapeutic approaches. The pathological progression of AP involves a complex interactive network of autophagic imbalance, uncontrolled inflammation, and impaired cell repair. This study systematically elucidates the central role of impaired autophagic flux in AP, untangles the causal relationship between lysosomal dysfunction (such as LAMP degradation and imbalance of cathepsin activity) and abnormal activation of pancreatic enzymes, and proposes that “autophagy activation with delayed flux” is a key feature of acinar cell injury in AP. This finding is consistent with previous research indicating that the accumulation of vacuoles resulting from autophagosome-lysosome fusion impairment is a common phenomenon in various experimental AP models.

The bidirectional regulatory characteristics of the PI3K/AKT/mTOR pathway are a critical aspect to consider in AP treatment. Our study reveals that this pathway may exhibit opposing effects at different stages of AP: during the acute phase, inhibiting the pathway can activate autophagy and reduce pancreatic enzyme activation, while activating the pathway during the repair phase promotes acinar regeneration and inflammation resolution. This suggests that interventions targeting PI3K/AKT/mTOR require dynamic regulation based on the disease phase. For instance, early use of wortmannin to inhibit PI3K can block abnormal activation of pancreatic enzymes mediated by CTSB, while later activation of the pathway through IGF-1 may accelerate tissue repair. This phase-dependent therapeutic strategy offers new insights for optimizing clinical medication regimens. Future research could further develop smart responsive delivery systems (such as pH-sensitive exosomes) to release PI3K inhibitors (like wortmannin) in the acidic microenvironment of the acute phase and IGF-1 activators during the repair phase.

With the extensive research on miRNAs, we have discovered that miRNA regulatory networks exhibit significant bidirectional plasticity in AP. Pro-inflammatory miRNAs (such as miR-155 and miR-216a) exacerbate autophagic imbalance by activating PI3K/AKT/mTOR or inhibiting PTEN, while anti-inflammatory miRNAs (like miR-148a and miR-20b-5p) alleviate inflammatory damage by inhibiting NF-κB/STAT3 signaling. Notably, molecules like miR-141 can both inhibit autophagosome formation (via the HMGB1/Beclin-1 axis) and reduce inflammatory responses, making them promising candidates for AP gene therapy due to their multi-target characteristics. However, organ-specific delivery and off-target effects of miRNAs remain major obstacles for clinical application.

MSCs and their exosomes demonstrate significant advantages in AP treatment due to their unique “multi-target synergistic regulation” properties. They can simultaneously target autophagy, inflammation, and regeneration pathways by delivering functional miRNAs. For example, miR-29a-3p carried by exosomes can inhibit the HMGB1/TLR4/AKT axis to reduce myocardial injury, while miR-216a-5p induces macrophage phenotype transformation through the TLR4/NF-κB and PI3K/AKT pathways, alleviating systemic inflammatory responses. This “cell-exosome-miRNA” cascade model overcomes the limitations of single-target interventions. Nevertheless, current research on the relationship between miRNAs and AP progression is still in its infancy, and studies examining the relationships between miRNAs, autophagy, and organelles in AP are scarce. Therefore, more systematic and in-depth investigations are needed to explore these relationships.

In conclusion, the therapeutic strategy for AP is shifting from single anti-inflammatory approaches to multi-target synergistic interventions. By integrating the restoration of autophagic homeostasis, inhibition of inflammatory pathways, and promotion of cell regeneration, precision and personalized treatment for AP may be achieved in the future. To achieve this goal, clinical translation can be advanced in stages. In Phase I trials, the pharmacokinetics and safety of exosomes are evaluated. In Phase II trials, relevant biomarkers are screened based on the subtypes of AP (such as biliary-derived and hyperlipidemia-induced). In Phase III trials, a multicenter study is conducted in combination with protease inhibitors to verify the efficacy of the trifecta therapy of “anti-inflammation - autophagy repair - regeneration”. This process not only provides a clear direction for AP research but also offers a reference and insight for interdisciplinary treatment strategies for other autophagy-related diseases.
